# Stochasticity in genetics and gene regulation

**DOI:** 10.1098/rstb.2023.0476

**Published:** 2024-04-22

**Authors:** Veronica van Heyningen

**Affiliations:** ^1^ UCL Institute of Ophthalmology, University College London, London, EC1V 9EL, UK; ^2^ MRC Human Genetics Unit, Institute of Genetics and Cancer, University of Edinburgh, Edinburgh EH4 2XU, UK

**Keywords:** stochastic events, genetic mechanisms, developmental noise, differentiation

## Abstract

Development from fertilized egg to functioning multi-cellular organism requires precision. There is no precision, and often no survival, without plasticity. Plasticity is conferred partly by stochastic variation, present inherently in all biological systems. Gene expression levels fluctuate ubiquitously through transcription, alternative splicing, translation and turnover. Small differences in gene expression are exploited to trigger early differentiation, conferring distinct function on selected individual cells and setting in motion regulatory interactions. Non-selected cells then acquire new functions along the spatio-temporal developmental trajectory. The differentiation process has many stochastic components. Meiotic segregation, mitochondrial partitioning, X-inactivation and the dynamic DNA binding of transcription factor assemblies—all exhibit randomness. Non-random X-inactivation generally signals deleterious X-linked mutations. Correct neural wiring, such as retina to brain, arises through repeated confirmatory activity of connections made randomly. In immune system development, both B-cell antibody generation and the emergence of balanced T-cell categories begin through stochastic trial and error followed by functional selection. Aberrant selection processes lead to immune dysfunction. DNA sequence variants also arise through stochastic events: some involving environmental fluctuation (radiation or presence of pollutants), or genetic repair system malfunction. The phenotypic outcome of mutations is also fluid. Mutations may be advantageous in some circumstances, deleterious in others.

This article is part of a discussion meeting issue ‘Causes and consequences of stochastic processes in development and disease’.

## 1. Introduction

The amazing thing about development is not that it sometimes goes wrong, but that it ever works. Successful development from fertilized egg to functioning multi-cellular organism requires precision. It may seem counterintuitive, but precision can only be achieved through plasticity. Decision-making based on stochastic variability is used repeatedly to progress through early development and differentiation. The pre-ordained deterministic role of genes and genetics has been frequently cited as an adverse constraint on adaptability. However, many random stochastic events contribute to the final outcome of gene-regulated processes. Stochastic events are also essential for generating the key substrate for selection of evolutionary innovation. Here, we explore some of these stochastic processes in biology. Frequently, these processes comprise obligatory steps allowing development to progress, but some are implicated in adverse effects leading to disease.

## 2. Progressive developmental cell-fate decisions require stochastic noise

As the fertilized egg undergoes initial cell divisions, ‘identical’ daughter cells are produced. Even at this stage transcriptional variability is present—such noise is an inherent component of biological systems [1–3]

At some point differentiation must be initiated and distinct cell types, capable of fulfilling different roles, must emerge [4,5]. Early developmental multi-potentiality is progressively constrained in an orderly manner to allow the predetermined sequential appearance of different cell types and tissues. Unexpectedly, orderly progression emerges from a noisy, somewhat disordered system. Stochastic cell-to-cell variability of gene expression is required for the establishment of cell identity. The fortuitous (transient) patterning, characterized by a quantitatively and qualitatively variable spectrum of gene expressions, allows the identity of the appropriate cell state to be locked in. Subsequent signalling between the selected cell type and its neighbours helps to confirm the new identity and further cell types with distinct molecular signatures can then emerge from the unselected cell pool. This general pattern of progressive development is observed in all multi-cellular systems. Recently, *in vitro* culture of a variety of stem cell types has enabled detailed observation of the principles and mechanisms involved [5,6].

It has been established for some time that transcription and translation do not progress at uniform rates, but in stochastic bursts [7]. Currently, specific RNAs are more readily assayed than specific proteins, so we know more about transcriptional than translational bursting [6]. Some of the randomness in transcriptional timing is imposed by factors such as the time required for RNA polymerase to traverse different length genes, particularly at times of rapid cell division when transcription is inhibited during DNA replication [8]**,** so only shorter genes get transcribed in rapidly dividing cells. However, the orderly emergence of the huge spectrum of different cell types, which can now be defined molecularly through single-cell transcriptomic analysis, is triggered and supported by the stochastic variation present from zygote formation to the emergence and maturation of the fully formed multi-cellular, multi-organ individual [9].

## 3. Partitioning of mitochondrial variants at oogenesis and during all cell divisions from zygote formation onwards

Multiple stochastic events are associated with mitochondrial inheritance and function, both very early in the life cycle at oogenesis, and during somatic cell division when these organelles have to partition between dividing daughter cells [10–12].

Mitochondria, the energy factories of cells, encode some of their essential subcellular machinery in mitochondrial DNA (mtDNA). The 16 kb circular mtDNA encodes 37 genes, mainly implicated in mitochondrial ribosomal function (22 tRNAs and 2 rRNAs). Only 13 genes encode protein components of oxidative phosphorylation, which constitutes the key part of mitochondrial function. Most mitochondrial proteins are nuclear encoded. Mutations, both in organellar- and nuclear-encoded mitochondrial genes, may seriously affect mitochondrial function, and, if uncovered by mitochondrial partitioning, may lead to distinct severe diseases [13]. Disease penetrance is likely to be observed when 70–80% of the mtDNA copies in a cell carry the deleterious mutation [12,14].

With 1000–10 000 copies of proliferating mtDNA molecules in most human cells, it is not surprising that random mutations in mtDNA arise frequently. Almost all cells are therefore expected to carry variant mitochondrial sequences and are said to be heteroplasmic for mtDNA. The variants may be functionally deficient, and some very severe diseases are caused by mtDNA mutations. Because the partitioning of mtDNAs during somatic cell division is initially a random process, but with some subsequent selection [15], the severity of mtDNA-linked diseases varies greatly even within families, between tissues, and may evolve within an individual during their lifetime [13]. Novel mutations can arise throughout life and mitochondrial dysfunction is implicated in many age-related degenerative diseases [16,17] and also in cancer [12].

Mitochondria are inherited solely through the maternal line, via the oocyte, because mitochondria are totally excluded from mature sperm. During the generation of each oocyte, there is a bottleneck, with stochastic and some selective loss of mitochondrial representation because the number of organelles that can be passed into the final oocyte produced is much reduced from 10 000, present in the precursor cell, to 1000 or fewer at meiosis [12]. There is both stochastic loss and some selection against deleterious variants with damaged capacity for oxidative phosphorylation [11,15]. Subsequently, mtDNAs are re-amplified to much higher numbers, up to 100 000 in the mature oocyte. Occasionally apparent paternal mtDNA inheritance has been suggested by family studies, but on fuller investigation this has now been shown to be due to rare stochastic insertion of mtDNA into the nuclear genome [18].

Interestingly, although the numerical reduction of mitochondria at oogenesis, and during partitioning in somatic divisions, is initially stochastic for sequence retention, there is inevitable selection for functional oxidative capacity since critical components of the mitochondrial protein assembly for this complex are encoded by the organellar DNA. Thus, cells with more functionally intact sequences will survive preferentially.

## 4. Random X-chromosome inactivation in XX individuals

The top-tier overriding sex determination mechanism in mammals is by chromosomal constitution. Females have two X-chromosomes (XX) and males an X and a male-determining Y (XY). This constitution is established at fertilization, so that post-meiotic sperm would normally be 50:50 X-bearing to Y-bearing, so approximately equal numbers of male and female embryos are produced by random fertilization. Gene dosage control is critical for correct functioning of many genes and is one of the reasons cited for the observed random inactivation of one of the two X chromosomes in XX individuals of all eutherian mammals [19,20]. X chromosome inactivation (XCI) arises by an epigenetic process involving the production and persistence of a long non-coding RNA, named XIST (X inactive-specific transcript). XIST RNA coats the inactivated X-chromosome. Though XIST is used in XCI by all eutherians, the precise mechanisms deployed are variable, but a unifying regulatory system is emerging for initiating and maintaining random XCI [21,22]. As illustrated by studies in the mouse, the chromatin organization of the active and inactive X become distinct [23]. The precise timing for the initiation of inactivation is thought to be very early, taking place in the epiblast, before implantation [19]. This timing and randomness can now be assessed by single-cell RNA expression studies in different tissues [24]. Such analysis also provides new data on the developmental timing of different tissues.

As a result of this developmentally very early random XCI, all tissues in female mammals are mosaic for the two alternative X-chromosomes in the active state. Where there is heterozygosity for functionally distinct alleles, mosaicism leads to phenotypic differences between neighbouring cells and tissues, depending on which chromosome is inactivated. It should be noted that approximately 20% of genes on the X-chromosome escape inactivation and remain biallelically expressed in each cell [19].

Ideally, when no deleterious mutations are present, the mosaicism is 50:50, but of course there is random fluctuation even for inactivation pattern [25] and a high proportion of females deviate from the 50:50 ratio. A normal distribution around the 50:50 peak is expected, as for heads and tails in coin tossing. In the absence of deleterious gene variants on the two X chromosomes, the deviation from 50:50 X-inactivation will have no observable phenotypic effect. However, skewed X-inactivation produced as a result of biological functional selection will also be observed when an XX individual carries a heterozygous deleterious mutation in an X-linked gene. Non-random inactivation for and against the deleterious allele may be observed [25–28]. Occasionally phenotypically affected heterozygous carrier females for X-linked recessive disease are identified. Conversely, skewed X-inactivation may be observed in some unusually mild cases of Rett syndrome involving mutations in the *MECP2* gene [29].

There is a disproportionately high presence of intellectual disability (ID) genes on the X-chromosome. Several different mechanisms can account for X-linked intellectual disability (XLID) observed in females who would generally be expected to be unaffected [26]. The presence of translocations involving the X-chromosome will usually lead to inactivation of the translocated X in XX individuals, because the translocation frequently has direct deleterious effects. If, in addition, there is a disease-causing variant at a locus on the active *non-translocation* X then the disease is expected to manifest. Such a situation has also been described for females with Duchenne muscular dystrophy [25,26]. Studying X-inactivation patterns in ID families with affected and unaffected female family members with XCI skewing in opposite directions, and often with affected males in the kindred, may identify variants whose causative nature was not initially recognized because it was also found in unaffected females [30].

## 5. Mutational mechanisms

Mutations arise constantly by many different mechanisms. Both germline and somatic mutations occur. At its simplest level, base sequence errors occur during DNA replication. Insertions of an incorrect nucleotide, or occasional omission or addition of one or a few nucleotides, are inevitable in systems which, despite being very precise, cannot be completely error-free and the surveillance/repair mechanisms sometimes fail. Most DNA sequence errors (single-nucleotide variants, SNVs) are considered to arise randomly in the genome, though recently the complete randomness of the process has been questioned [31] when a single site is considered. Certainly, transitions (purine–purine or pyrimidine–pyrimidine changes) are more common than transversions (purine–pyrimidine or vice versa) [32], particularly where CpG sequences are present in DNA.

Some SNVs arise through chemical or physical (e.g. UV) alteration of bases leading to altered nucleotide pairing at subsequent replication [33], or by exposure to other environmental agents such as natural radiation, illustrated by elevated lung mutation rates seen in granite geological regions where higher radon levels are associated with increased lung cancer levels [34]. Damage by these mechanisms has a strong stochastic component.

Throughout the genome, larger structural changes (structural variants, SVs) arise randomly during meiotic recombination and segregation, although with some spatial constraints. Deletions, duplications, inversions and chromosomal translocations are all relatively common and frequently lead to deleterious phenotypic outcomes. Multiple mechanisms are responsible for the genesis of such structural variations. Data have been collected in an open database [35]. Also included in the database are outcomes from one mechanism that we have not yet mentioned. Many human variants arise when mobile genetic elements are released and insert randomly into another part of the genome [36]. Additional critical developmental roles for such transposable elements in early embryos are still being uncovered [37]. The recently developed early embryo models from *in vitro* differentiation of stem cells have contributed to these new insights.

Deletions and duplications, especially those arising *de novo*, are often associated with disease. Copy number variants (CNVs) have recently been associated with many dosage-sensitive regions throughout the genome [38,39]. Somatic genomic replication also poses hazards for genome integrity with random double-strand breaks fairly frequently failing to be fully repaired in neuronal tissue as individuals age. Structural variants arise and accumulate in this situation, and are often associated with neurodegeneration [40].

Huge numbers of SNVs are documented in most organisms, including humans. Many are common single-nucleotide polymorphic (SNP) variants which may have a role in phenotype modulation. Some are directly disease-causing, usually rare, variants. Historically, most of the latter have been documented in the coding region of genes, but with more sequence data availability SNVs [41,42] and SVs [43–45] with phenotypic effects are being defined in non-coding regions where alterations in gene regulation are increasingly demonstrated to cause disease. All types of genomic rearrangements may disrupt protein-coding [46] or untranslated RNA transcription units [47] or gene regulatory regions in non-coding domains [48,49]. The full spectrum of variants may be associated with disease, including some classified as developmental anomalies. Conversely, variants are also potential targets for evolutionary selection if the associated phenotypes confer a selective advantage.

Errors in splicing are often caused by random mutations, as discussed above, perturbing functional splicing signals. Abnormal splicing is a frequent cause of disease [50]. Many of these are stochastic. In addition there is evidence for random splicing events taking place [51]. Multi-exon genes are almost universally subject to different alternative splicing patterns [52]. The pattern of alternative splicing is frequently tissue-specific. Many RNA molecules are subject to surveillance mechanisms such as nonsense-mediated decay (NMD) [51] to ensure that functionally intact RNAs are delivered to fulfil their roles.

In addition to disease-causing splicing variation, random splicing events and exon shuffling are also important mechanisms permitting evolutionary change and adaptation [52,53].

## 6. Meiotic segregation

In a diploid organism's life cycle meiosis only takes place once. Meiosis is the complex multi-stage ‘reduction division’ in which the diploid organism produces haploid germ cells that will combine at fertilization to restore the zygote to the diploid state for the next generation. This diploid to haploid cycling with each generation is critical to permit each parent to contribute equally to the next generation [54]. The meiotic division process must be very precise to ensure that a full complement of genetic material is passed to each egg and sperm produced. Accurate pairing—synapsis—of homologous parental chromosomes is aided by compulsory exchange of DNA between pairing homologues, by crossing-over or chiasma formation. Such cross-over events generally take place at least once on each chromosome arm. The position of meiotic cross-overs is not random. They tend to arise away from centromeric constraints and are associated with recognizable sequence motifs [55]. There is also interference between neighbouring cross-overs: they cannot occur too close to each other, but within the permissive regions meiotic exchange is random. Meiotic exchanges between homologues are resolved by well-studied molecular mechanisms, involving multiple DNA exchange and repair proteins [54,56–58].

Following the meiotic pairing and exchange, or gene conversion events, four different recombined homologous chromatids are produced for each paired chromosome. The chromatids reduplicate so chromosomes are restored. Each meiotic homologue is an obligatory mixture of grandmaternal and grandpaternal DNA [54,57]. Each male meiotic event gives rise to four haploid male germ cells, but female meiosis only produces one oocyte and three polar bodies [59]. When germ cells are produced, there is independent random assortment for the four possible homologous products for each of the 22 autosomes. Independent random assortment is a major principle of Mendelian genetics [60]. There is excellent evidence from large-scale allelic segregation studies at many loci that this random assortment of meiotic products is the mechanism that prevails [61]. Obviously, there may appear to be segregation anomalies due to selection at loci where alleles deleterious for survival arise. Such variants may affect gamete survival but, more likely, selection manifests post-fertilization, prenatally or postnatally, so that the predicted random-mating Mendelian ratios are not observed at the point of counting. Additionally, there is evidence for segregation distortion [62].

## 7. Inheritance of X and Y chromosomes

In male meiosis, X and Y chromosomes have a short obligate pairing region where exchanges can take place. This region is called the pseudo-autosomal region because genes residing here do not show classical X-linked inheritance patterns [63]. The X and Y chromosomes segregate randomly to male germ cells. Following random fertilization by X- and Y-bearing sperm, very close to equal frequency of male and female offspring are born. The random mating ratios are certainly observed at the population level, although individual X or Y chromosomes carrying alleles deleterious for (mainly male) survival may be selected against in individual families. One obvious situation would be where alleles deleterious for male fertility arise and cannot be passed on by the carrier.

## 8. Genealogical and genetic ancestry

**Figure 1.**
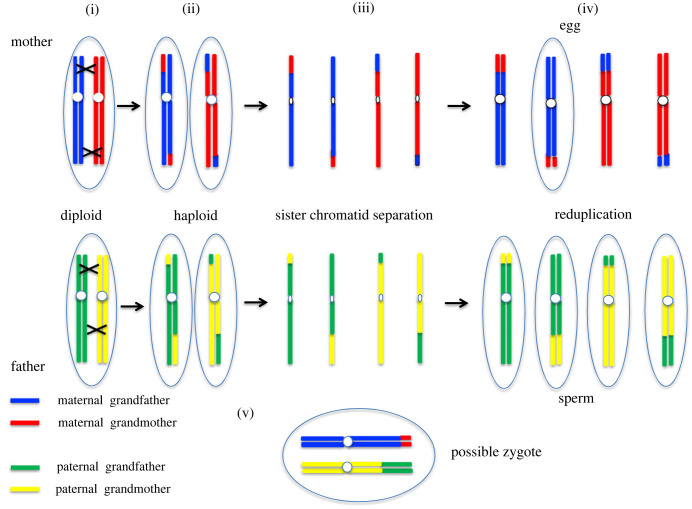
Chromosome segregation at meiosis: loss of grandparental information. Meiosis and meiotic segregation in the germline. Exploring the loss of grandparental information from one generation to the next. Following the fate of just one homologous pair of chromosomes as haploid germ cells are produced in meiosis, ready for recombination at fertilization. The four grandparental chromosomal contributions are distinguished by colour. The stochastic loss of grandparental information can be followed. (i) Pairing of the two duplicated homologues at meiosis I, reciprocal exchange of genetic material between grandparental homologues. Crossing-over takes place at partially random sites (with some constraints). (ii) Segregation of the recombined homologues, (iii) separation of sister chromatids, (iv) reduplication of chromatids to restore chromosome homologue. Of the four final meiotic products, only one oocyte is produced, but potentially four spermatocytes. (v) Following final maturation of the germ cells fertilization takes place with a large number of possible zygotic outcomes showing different levels of grandparental representation for each of the 22 autosomes. After several generations, the contribution from an ancestor may be completely lost.

One interesting rarely discussed aspect of exploring an individual's ancestry is that there are two levels at which this concept can be discussed. One is a historical feature in which a family tree can be built up from knowledge of past mating details. However, in the current era of polymorphic variant analysis and genome sequencing, we can also deduce the current genetic heritage of an individual [64]. With more available information it becomes clear that with stochastic segregation of ancestrally recombined chromosomal homologues, individuals may lose all DNA contribution for a relatively recent ancestor (from seven or more generations back). This is not surprising. In each generation, at every locus, only two of the four grandparental alleles pass into each sperm or egg. Therefore, we lose ancestral genetic information constantly (figure 1). Statistically, as a result of such constant random loss at all loci, all contributions from a far-back ancestor may be lost on all chromosomes in an individual.

## 9. Random events in developmental systems

Neuronal plasticity is one of the most frequently examined phenomena where developmental fluctuation is required to produce adaptable, dynamic, but precise networks. Stochastic events often trigger the making of secure neural connections, which are strengthened by eliciting successful signalling pathways that are reinforced by repeated activity. Such a process is implicated in the wiring of the retinotectal map in the brain. Coordinated waves pass across the developing retina even *in utero*, and recurrent activity confirms the functioning connections to the tectal regions of the brain [65,66]. In this way, a finely detailed representative retinotectal map is produced and maintained through recurrent reinforcing activity. More generally, stochastic activity of neural networks is critical for the development and maintenance of fully functional neural connectivity in the brain [67].

Photoreceptors for precision daylight vision and colour perception arise in the central area of the human retina. Which of the three wavelength receptors (Long, Medium and Short) is expressed in each cone as it develops is a stochastic event, and as a result the central retina is a random mosaic of red, green and blue cones [68]. As with many stochastic processes in biology, there are also additional layers of genetic regulatory control, before and/or after the stochastic event.

Random activation of individual G-protein coupled olfactory receptor (OR) genes is critical for precise olfactory function. A single allele of one receptor gene is expressed in any given sensory olfactory neuron in the olfactory epithelium. There are a thousand or more distinct functional OR genes in mammals like rodents with sensitive olfactory function. Humans have fewer functional genes, though quite a few pseudogenes are to be found in their genome. The control system for expressing only one gene in one epithelial cell is complex. These epithelial cells then signal to the neural system when activated by a very narrow spectrum of odorant molecules that trigger that particular receptor. Activity-dependent connections are then set up, linking to a specific site in the olfactory glomeruli [69,70]. The different ORs that detect chemically related odorants wire to neighbouring sites in the glomeruli of the olfactory bulb. These events are reiterated many times to produce a regionally connected network of functionally related receptor expression. This complex system is still under ongoing study [71].

## 10. Immune response

Major agents of the adaptive immune system in vertebrates are the lymphocytes (white blood cells). When there is an immunological challenge, such as infection by bacteria and viruses, two distinct lymphoid cells, the B-cells and the T-cells, can contribute to the immune response. B-cells produce antibodies which are proteins that can recognize and help eliminate antigens. T-cells provide a cellular response which can act independently or cooperatively with B-cells. Both cell types need to recognize a very wide spectrum of antigens in different ways and this very broad response capacity is generated by rearrangement of genes encoding complex recognition proteins. The generation of a diverse spectrum of antibody molecules and also T-cell antigen receptors is achieved by similar genomic recombination involving a large number of V (variable) D (diversity) and J (joining) segments and specific recombinase enzymes [72]. This system can randomly generate a huge number of novel potential recognition-site sequences. The cells where recombination has taken place will have their new recognition site ‘tested’ when an antigen is encountered. If there is sufficiently strong protein–protein interaction the cell will be selected for. Non-functional rearranged cells die. In antibody-producing B-cells further variability can be conferred on the selected cells with the help of additional stochastic processes that are able to generate slight changes in the protein to improve its affinity for the antigen. This step is termed affinity maturation [73–75]. The stochastic nucleotide change function is essentially a mutagenic step. There are multiple safeguards to constrain it to this activity, but aberrant functioning of the system is implicated in oncogenic events.

The generation of almost limitless protein sequence variation in the variable portion of the B-cell receptor/antibody or the T-cell antigen-receptor is followed by very strong selection against the combinations that do not confer high-affinity binding capacity to the antigen. This pattern of random trial-and-error variation, followed by the survival of the fittest cell type is a frequently encountered mechanism when exploring stochasticity in biology.

## 11. Phenotypic variability

In the first section of this paper, stochastic transcriptional fluctuation was described as a trigger for initiating robust precise developmental progression. Constraining function is also cited as *one* role for stochasticity by MacNeil & Walhout [76]. However, the association of stochastically variable gene expression with phenotypic variability is a second major theme developed by MacNeil and Walhout and others [77]. The existence of physiological variability is of major importance for environmental adaptation and survival of complex organisms. Many aspects of phenotype are affected by random fluctuation of the biochemical background. A distinct mechanism that has been much studied recently is epigenetic change that is frequently triggered by stochastic variation and random events [78]. Epigenetic changes are mitotically heritable and occasionally also carried from one generation to the next, but do not involve DNA sequence change. Epigenetic changes can involve DNA and/or histone modification changes and non-coding RNA interactions. Stochastic conformational changes affecting chromatin structure may also contribute to variable gene expression [79].

Disease-associated phenotype variation is often seen with well-defined causative mutations. Differences between family members carrying the same mutation are frequently due to background genetic differences between family members. Temporal fluctuation of disease phenotype within an individual may be the result of stochastic gene expression. This may be the underlying trigger for the onset of attacks in genetic epilepsies or migraine, or for variable severity of inflammatory diseases within individuals, but literature on this was not readily found.

Distinct unilateral individual phenotypes in genetic disease of paired organs is very likely caused by localized stochastic differences in gene expression, possibly occurring at a critical time in development. Such unilaterality is frequently seen in developmental eye anomalies and also some limb phenotypes. We reported a striking occurrence of unilateral anophthalmia, in two different members of family 3432, associated with dominant (haploinsufficient) protein truncating mutation in the *SOX2* gene [80]. Positional fluctuation in the level of functional SOX2 protein is the simplest explanation for this observation. Many haploinsufficiency diseases involve genes, often transcription factors, that fulfil key roles requiring a specific threshold level of activity. Quite small variation in expression level can tip the balance between functionality and anomaly.

## 12. Epilogue

We have discussed how stochastic or random events exert their influence in diverse ways. They can confer order but can also generate necessary variability or plasticity.

Transcriptional noise triggers ordered development along predetermined precise paths from fertilized egg to complex embryo and fetus. Conversely, stochasticity can confer phenotypic variability, allowing organisms to adapt to, and survive in, changing environments. Both genetic and environmental effects modulate the outcomes of stochasticity.

Random events such as X-inactivation, meiotic chromosome segregation in the germline, mitochondrial bottlenecks and partitioning as well as the constant generation of DNA level variation (mutations) provide opportunities for selection to drive evolution, enabling adaptation to environmental change.

The wide-ranging roles of stochastic events in genetic processes and in development are not widely recognized by scientists, and to an even lesser extent by the general population. Recognition that random events are inbuilt and often essential components of biological mechanisms is important for countering the concept of relentless genetic determinism. Along with environmental factors, stochastic events in multiple processes throughout the lifecycle play a critical role in phenotype development and maintenance. Philosophically and emotionally it is important to acknowledge that many adverse, as well as some neutral or beneficial, life events arise through random chance.

## Data Availability

This article has no additional data.
